# Progressive Fibrosing Interstitial Lung Diseases: A Current Perspective

**DOI:** 10.3390/biomedicines9091237

**Published:** 2021-09-16

**Authors:** Carlo Albera, Giulia Verri, Federico Sciarrone, Elena Sitia, Mauro Mangiapia, Paolo Solidoro

**Affiliations:** 1 Department of Medical Sciences, School of Medicine, University of Turin, SC Pneumologia U, 10124 Turin, Italy; federico.sciarrone@unito.it (F.S.); elena.sitia@unito.it (E.S.); paolo.solidoro@unito.it (P.S.); 2 Ciità della Salute e della Scienza, Molinette Hospital, SC Pneumologia U, 10124 Turin, Italy; gverri@cittadellasalute.to.it (G.V.); mmangiapia@cittadellasalute.to.it (M.M.)

**Keywords:** antifibrotic therapy, fibrosis, idiopathic pulmonary fibrosis, interstitial lung disease, nintedanib, pirfenidone, progressive fibrosing interstitial lung disease

## Abstract

Interstitial lung diseases (ILDs) are a large and diverse group of rare and chronic respiratory disorders, with idiopathic pulmonary fibrosis (IPF) being the most common and best-studied member. Increasing interest in fibrosis as a therapeutic target and the appreciation that fibrotic mechanisms may be a treatable target of IPF prompted the development and subsequent approval of the antifibrotics, pirfenidone and nintedanib. The management of ILDs has changed considerably following an understanding that IPF and some ILDs share similar disease behavior of progressive fibrosis, termed “progressive fibrosing phenotype”. Indeed, antifibrotic treatment has shown to be beneficial in ILDs characterized by the progressive fibrosing phenotype. This narrative review summarizes current knowledge in the field of progressive fibrosing ILDs. Here, we discuss the clinical characteristics and pathogenesis of lung fibrosis and highlight relevant literature concerning the mechanisms underlying progressive fibrosing ILDs. We also summarize current diagnostic approaches and the available treatments of progressive fibrosing ILDs and address the optimization of treating progressive fibrosing ILDs with antifibrotics in clinical practice.

## 1. Introduction

Fibrosis refers to an exaggerated accumulation of extracellular matrix proteins in response to inflammation and injury [1,2]. This process, which can be regarded as unregulated and prolonged wound healing, is often the underlying mechanism of organ damage and failure [1]. In recent years, fibrosis has attracted considerable interest as a therapeutic target [1]. Pirfenidone, an inhibitor of transforming growth factor-ß (TGFß), and nintedanib, a tyrosine kinase inhibitor, were the first drugs to be approved by the European Medicines Agency (EMA) and the Food and Drug Administration (FDA) for the treatment of idiopathic pulmonary fibrosis (IPF), a chronic and fatal interstitial lung disease (ILD).

ILDs form a large and heterogeneous group of rare and chronic respiratory disorders that present with damage to the lung parenchyma, usually caused by a combination of inflammation and fibrosis [3]. IPF is the most common and best-studied member of this group [4]. It is characterized by progressive pulmonary fibrosis and a loss of lung function, and it has poor prognosis with a median survival of 3 years from diagnosis [5]. IPF is generally regarded as the prototype of fibrosing ILDs [4]. While not all ILDs are associated with progressive fibrosis, those that develop this phenotype possess a clinical course similar to IPF [3,6]. 

ILDs have traditionally been treated with off-label corticosteroids and/or immunosuppressive therapies, with variable effectiveness depending on the disease. In particular, the PANTHER trial, showing that the combination of azathioprine, prednisone and N-acetylcysteine worsened the outcomes of IPF patients, provided compelling evidence that inflammatory and immune responses may not be the optimal target [7]. The development of antifibrotics, pirfenidone and nintedanib, was prompted by the recognition that fibrotic mechanisms, rather than early pathogenic events, may be a treatable target of IPF [8]. In their registration trials, both drugs significantly reduced IPF progression compared with placebo [9,10,11]. 

In recent years, the similar disease behavior shared by IPF and progressive fibrosing ILDs has resulted in the hypothesis that antifibrotic treatment may be beneficial in other ILDs characterized by the progressive phenotype [8]. This hypothesis has been confirmed in the phase 3 INBUILD clinical trial with nintedanib in patients with non-IPF ILDs [12]. The phase 3 SENSCIS study with nintedanib also reached its primary endpoint, supporting this hypothesis, but the patient population included patients at risk of ILD progression as well as those with a progressive phenotype [13]. The phase 2b study with pirfenidone in unclassifiable ILD (a subset of ILDs that are unable to be classified according to the current diagnostic framework, see O’Callaghan et al. for a recent comprehensive review [14]) did not reach its primary endpoint; hence, its results are only suggestive and not conclusive [15]. 

The results of these trials showed that the antifibrotic, nintedanib, significantly reduced the decline of lung function compared with placebo and to a similar extent to that observed in patients with IPF [12,13], resulting in new licensed indications of antifibrotics. Nintedanib is currently approved by the FDA and EMA for the treatment of IPF, systemic sclerosis-associated ILD and ILDs with a progressive phenotype [16,17]; pirfenidone is approved by the FDA and EMA for the treatment of IPF [18,19].

While the availability of targeted drugs is no doubt a major advance in the management of progressive fibrosing ILDs, several practical issues remain challenging, including the diagnosis and classification of these conditions; their variable clinical course; the lack of validated biomarkers for the prediction of disease progression; and the lack of a clear definition of a progressive fibrosing phenotype. The aim of this narrative review is to summarize current knowledge in the field of progressive fibrosing ILDs and to suggest methods for improving the management of these rare conditions with the new drugs. To this purpose, we will first discuss the most relevant literature concerning the mechanisms underlying progressive fibrosing ILDs; we will then discuss current diagnostic approaches and the available treatments. Finally, we will address the optimization of the treatment of progressive fibrosing ILDs with antifibrotics in clinical practice.

## 2. Clinical Characteristics and Pathogenesis of Lung Fibrosis

### 2.1. Definition and Diagnosis

IPF, the prototype of fibrosing ILDs, is defined in a joined guideline by the American Thoracic Society (ATS), the European Respiratory Society (ERS), the Japanese Respiratory Society (JRS) and the Latin American Thoracic Association (LATS) as a “specific form of chronic, progressive fibrosing interstitial pneumonia of unknown cause, occurring primarily in older adults, and limited to the lungs.” [20]. It is associated with the histologic and/or radiologic pattern of usual interstitial pneumonia (UIP). IPF is a fatal disease with variable and unpredictable natural history. Disease progression is evidenced by increasing respiratory symptoms, worsening pulmonary function, progressive fibrosis on high resolution computed tomography (HRCT), acute respiratory decline or death [20]. 

A multidisciplinary approach involving pulmonologists, radiologists and pathologists improves the accuracy of diagnosis [20,21]. The diagnosis of IPF requires the exclusion of known causes of ILD, such as the exposure to triggers, the presence of connective tissue disease or drug toxicity and a pattern of UIP on HRCT [20,21]. According to a White Paper published by the Fleischner Society, a confident diagnosis of IPF can be made when HRCT shows a pattern of definite or probable UIP [22]. Lung biopsy should be considered when the HRCT pattern does not show definite or probably UIP [22]. When lung biopsy cannot be performed, a working or provisional diagnosis of IPF can be made after multidisciplinary evaluation. The usefulness of a working diagnosis was also acknowledged in a recent Delphi survey among international ILD experts, and the ultimate goal was to improve the clinical utility of future guidelines for the diagnosis and management of IPF [23]. This approach should help patients receive adequate treatment in countries where a diagnosis of IPF is required for the prescription of antifibrotics [23].

### 2.2. Risk Factors and Genetics

Although the etiology of IPF is unknown by definition, several risk factors have been identified, including aging, cigarette smoking, environmental exposures, viral infections and chronic tissue injury (e.g., gastroesophageal reflux) [20,24]. Genetic factors that predispose individuals to the development of IPF have also been described [20,25]. Familial forms of IPF exist, although they are rare and account for <5% of total cases [20]. The study of large affected families has resulted in the discovery of several genes associated with familial IPF and contributed to uncovering some of the mechanisms underlying fibrosing ILDs [25]. Several of these genes are related to the regulation of length and stability of telomeres, which are the ends of chromosomes that are crucial for chromosome integrity [25,26,27]. Pathogenic mutations in telomere genes can result in short telomeres and chromosome degradation, eventually resulting in fibrosis and organ dysfunction [27]. The reverse telomerase transcriptase (TERT) gene is the most frequently mutated telomere gene, with variants occurring in 20% of familial IPF [26].

Genome-wide association studies have identified single-nucleotide polymorphisms (SNPs) in patients with IPF [25]. In particular, polymorphisms in the promoter of MUC5B (rs35705950) and in an intron near the TOLLIP (Toll-interacting protein) gene (rs5743890) have been found to influence the risk of developing IPF [25,28]. MUC5B is a polymeric mucin important for airway clearance and function and immune homeostasis, while TOLLIP encodes a mediator of innate immune responses and airway host defense [25,28]. It is thought that the increased expression of MUC5B, induced by the SNP in the promoter region, may impair the defense mechanisms of the bronchial mucosa against external agents and, thus, alter the repair mechanisms of lung parenchyma [24].

The 2018 ATS/ERS/JRS/ALAT guideline for IPF diagnosis lists genetic markers among the topics requiring further research and points out that, although genetic variants contribute to the risk of developing IPF, their clinical relevance needs to be demonstrated in future studies [21].

### 2.3. Pathogenesis

Similar to other chronic diseases resulting in organ failure, IPF results from the combination of genetic susceptibility and environmental risk factors [24,29]. Despite great advances over the past two decades in the identification of pathways relevant to IPF, the pathogenesis of this condition is not well understood. The unregulated behaviors of alveolar epithelial cells and myofibroblasts are generally regarded as the key mechanisms underlying the development and progression of fibrosis [29,30]. 

The injury of alveolar epithelial cells is followed by a cascade of repair processes that involve an inflammatory response with the accumulation of lymphocytes and macrophages at the injured site and release of profibrotic mediators [1,24]. Fibroblasts proliferate and migrate to the site of injury and eventually differentiate to myofibroblasts [1,24]. Myofibroblasts constitute a heterogeneous cell population characterized by the ability to express contractile proteins, high levels of extracellular matrix proteins and fibrogenic cytokines [31]. In normal wound healing, once tissue damage has been repaired, repair processes are terminated and myofibroblasts are eliminated via apoptosis [1]. In fibrotic diseases, persistent and unregulated myofibroblasts that fail to undergo apoptosis contribute via excessive deposition of connective proteins to lung tissue structural and mechanical changes (remodeling), resulting in the loss of alveolar function [1,31]. The fibrosing process eventually becomes self-perpetuating, as increased lung tissue stiffness and damage further recruits and activates myofibroblasts [1].

## 3. Progressive Fibrosing Interstitial Lung Disease

The UIP pattern has always been indicative of a diffuse fibrosing pathology with a progressive trend regardless of the precise diagnosis [3,20,21,22,32]. That is, when a UIP pattern is present on imaging, the disease behaves similarly to a UIP, whether it is idiopathic or not. The aspect that can actually influence the behavior of the disease is the speed of worsening of involvement in UIP patterns such as in rheumatoid arthritis or non-specific interstitial pneumonia patterns of systemic sclerosis, which are generally less rapidly progressive compared with analogous idiopathic forms. 

A proportion of ILDs can have a disease course overlapping with that of IPF, with similar high morbidity and mortality [3]. A recent study based on data from the placebo arms of the INPULSIS (IPF patients) and INBUILD (patients with progressive ILDs other than IPF) trials and investigating the natural history of IPF and progressive non-IPF ILDs has shown similar outcomes [33]. INPULSIS patients with IPF and INBUILD patients with progressive non-IPF ILDs had similar rates of >10% decline in forced vital capacity (FVC) (48.7% and 48.9%, respectively) and mortality rates at 52 weeks (7.8% and 5.1%, respectively) [33]. The exact prevalence of progressive fibrosing ILDs is unknown. The recent PROGRESS study in a real-world cohort of patients with ILDs has identified a progressive phenotype in approximately 25% of patients with fibrosing ILDs other than IPF [34]. Based on the results of an international survey among clinicians managing patients with non-IPF ILDs, the proportion of patients diagnosed with non-IPF ILDs who develop a progressive fibrosing phenotype ranged from 18% to 32%. According to the survey, the time from symptom onset to death was 61–80 months in these patients [35]. The prevalence of progressive fibrosing ILDs in patients who attended two Italian referral centers from 1 January 2011 to 31 July 2019 was 31% (75 of 245 patients with non-IPF fibrosing ILDs) [36]. The median survival after the diagnosis of ILD progression was 3 years, with 2 year and 3 year mortality rates of 4% and 20%, respectively. 

In addition to IPF, the ILDs most likely to develop a progressive fibrosing phenotype include idiopathic non-specific interstitial pneumonia; unclassifiable idiopathic interstitial pneumonias; rheumatoid arthritis-associated ILD; systemic sclerosis-associated ILD; hypersensitivity pneumonitis; sarcoidosis; and ILDs related to other occupational exposures (e.g., asbestosis and silicosis) [3]. These diseases are rare [6], poorly investigated and difficult to diagnose (for a thorough review, the reader is referred to Cottin et al. 2018 [3]). Accurate diagnosis requires a multidisciplinary discussion between the pulmonologist, radiologist and pathologist, and it is crucial for therapeutic decisions [3]. As with IPF, a definite diagnosis cannot be made in many cases. As recommended by the Fleischner Society White Paper [22], a working diagnosis may be sufficient in such cases. In clinical practice, a working diagnosis of IPF or progressive fibrosing ILD appears to be a more than reasonable background and, hopefully, may be sufficient for the prescription of antifibrotic therapy. A recent survey involving 404 international respiratory physicians, who were asked to evaluate 60 cases of ILD, showed that antifibrotic therapy was prescribed in the absence of a lung biopsy when a working diagnosis could be made with a likelihood ≥70% [37].

The recognition that IPF and certain ILDs can share a behavior of progressive fibrosis—termed “progressive fibrosing phenotype”—despite distinct initial disease triggers has considerably changed the management of ILDs [29]. Grouping distinct disease entities under the concept of a progressive fibrosing phenotype has contributed to providing novel treatment options to patients affected by rare diseases that cannot be individually investigated in adequate trials for the lack of patients [29]. Currently, an exact definition of progressive fibrosing phenotype is not available. The patient inclusion criteria of the INBUILD trial investigating the efficacy and safety of the antifibrotic nintedanib in patients with progressive fibrosing ILDs other than IPF required disease progression despite standard treatment [12]. Progression was defined by a relative decline in FVC ≥ 10%; or a relative decline in FVC of 5–10% with worsening respiratory symptoms or increased fibrosis on HRCT; or worsening respiratory symptoms and increased fibrosis on HRCT [12]. 

An analysis of the INBUILD data showed that there are well-established parameters (i.e., pulmonary function test imaging symptoms) that can identify the trend in progression. Specifically, the inclusion criteria of the INBUILD trial were shown to be effective in identifying patients with fibrosing ILDs with a progressive phenotype [38]. 

Importantly, rapidly non-IPF progressive fibrosing ILD was described in three patients who were younger than the average patient with IPF, had received a non-IPF multidisciplinary team diagnosis and had a non-UIP pattern on HRCT [39]. This case series suggests that patients presenting with similar characteristics should be treated with antifibrotics, rather than immunosuppressive drugs, due to the potential development of a rapidly progressive ILD phenotype.

The clinical practice-oriented definitions of the progressive fibrosing phenotype recently suggested in a position paper from the Erice ILD working group are reported in Table 1 [40].

We have always been accustomed to try to exclude, with all rational efforts and according to good clinical practice, the secondary nature of the progressive disease of ILD. This is because, with our increasing knowledge on the pathogenesis of IPF, the evidence supported the antifibrotic treatment of IPF (nintedanib and pirfenidone) as the only effective treatment capable of improving outcomes. There is no evidence to support antifibrotics in the setting of a non-definitive diagnosis of IPF, despite it being clear that the fibrosis is the same as IPF, and the standard of care, therefore, includes steroids, immunosuppressants and cytotoxic agents.

With the evidence to date on nintedanib (SENSCIS and INBUILD), the scenario has changed. A diagnosis based on clinical behavior (i.e., progressive fibrosing interstitial disease) is sufficient for using an antifibrotic with similar success compared to that of IPF. It could be argued that the precise diagnosis is the first aim of the clinical procedure in order to prescribe suitable therapy. Indeed, the question arises as to whether our judgment of suitable therapy would remain the same if the results of SENSCIS and INBUILD had been available earlier. 

HRCT is important for the diagnostic evaluation of fibrosing ILDs, as reviewed by Torres and colleagues [41]. Moreover, the comparison of follow-up CT scans with initial CT scans is useful for identifying progressive fibrosing ILDs [42]. HRCT, however, may require fine-tuning and standardization of methods to quantify the involvement of the parenchymal muscle and to measure its variations in repeatable accessibility techniques. Indeed, this was identified in a Danish cohort study, which showed that a simple HRCT scoring system could be used to predict outcomes in fibrotic ILDs [43].

A 2018 review on the role of imaging in progressive fibrosing ILDs not only identified HRCT as central to the diagnosis in ILDs but also pointed out the need to identify new methods for image analysis, which quantify the extent and progression of fibrous pathologies using a machine learning-based approach [32]. The link between state-of-the-art imaging and physical semeiotics, which includes the historical heritage of pulmonology, was recently established in a proof-of-concept study of a machine learning-based approach to quantify fine crackles in the diagnosis of interstitial pneumonia [44]. This study reported that machine learning-based quantification of fine crackles, which are frequently heard in patients with ILDs, can predict the HRCT findings of lung fibrosis, thereby supporting the timely and sensitive diagnosis of ILDs.

Hence, an optimal approach to disease staging and outcome prognosis in fibrosing ILDs with a progressive phenotype may be achieved by combining semi-quantitative imaging techniques, based largely on visual analysis, with quantitative imaging coupled to machine learning [32].

Lung biopsy, alongside the development of less invasive procedures, is also useful in the diagnosis and assessment of the progressive fibrotic phenotype, as recently reviewed by Ravaglia and colleagues [45].

Several serum biomarkers have been tested for their ability to predict disease progression, but no molecule has been validated as a prognostic marker for use in clinical practice so far [40]. As for genetic variants, the SNP rs35705950 in the promoter region of MUC5B in IPF has also been shown to be associated with rheumatoid arthritis-associated ILD and in hypersensitivity pneumonitis [46,47]. Variants in genes related to telomere homeostasis have been identified in rheumatoid arthritis-associated ILD, systemic sclerosis-associated ILD and hypersensitivity pneumonitis [47,48,49]. The association between these variants and ILD development and progression remains to be defined.

Current understanding is limited regarding the pathogenesis of progressive fibrosing ILDs. Some ILDs are primarily fibrotic, with fibroblast dysfunction resulting in fibroblast proliferation and fibrosis, as discussed above for IPF. Other ILDs are predominantly inflammatory disorders but can shift, via mechanisms that are currently unknown, to a fibrotic pathway under certain conditions. Although the initial pathogenetic mechanisms are distinct between these two groups of ILDs, the processes resulting in self-sustaining fibrosis and organ damage are likely to be common [29,50]. 

## 4. Treatment of Progressive Fibrosing Interstitial Lung Disease

In the latest update of the ATS/ERS/JRS/ALAT guideline for IPF treatment published in 2015, nintedanib and pirfenidone, along with antacid therapy, are the only drugs to be recommended for the treatment of IPF (conditional recommendation for all three therapies) [51]. Nintedanib is an intracellular inhibitor of several tyrosine kinases that regulate multiple growth factor receptors implicated in the pathogenesis of fibrosis in ILDs, including vascular endothelial growth factor, fibroblast growth factor and platelet-derived growth factors (PDGF) [16]. The mechanism of action of pirfenidone is not fully understood. Pirfenidone has both antifibrotic and anti-inflammatory properties; in in vitro and animal models of pulmonary fibrosis, it has been shown to reduce fibroblast proliferation, the release of fibrosis-associated proteins and cytokines and the excessive production and accumulation of extracellular matrix in response to TGF-ß and PDGF [18]. Efficacy and safety of the two antifibrotics have been demonstrated in several large phase three, randomized, controlled clinical trials in patients with IPF [9,10,11]. 

Currently, there are no treatment guidelines issued by a scientific society to guide clinicians in the treatment of progressive fibrosing ILDs other than IPF. Treatment of these conditions usually involves corticosteroids or immunosuppressants, including azathioprine, mycophenolate mofetil and cyclophosphamide [3]. However, these medications have not been investigated in randomized controlled trials in progressive fibrosing ILDs, with the exception of systemic sclerosis-associated ILD [3]. In this condition, cyclophosphamide and mycophenolate mofetil have been shown to improve FVC versus placebo [3].

Given the general lack of evidence supporting immunomodulatory treatments for progressive fibrosing ILDs other than IPF, as well as the lack of efficacy of these treatments in many patients, the results of the trials investigating antifibrotic drugs in patients affected by these conditions [12,13,15] are considered a breakthrough in the field of ILD. The INBUILD trial randomized 663 patients with fibrosing ILDs other than IPF to nintedanib or placebo [12]. The fibrosing ILDs were as follows: chronic hypersensitivity pneumonitis (26%), autoimmune ILD (26%), idiopathic non-specific interstitial pneumonia (19%), unclassifiable idiopathic interstitial pneumonia (17%) and other ILDs (12%) [52]. All patients had progressive disease in the past 24 months, despite receiving treatment. The primary endpoint was the annual rate of decline in the FVC, assessed over 52 weeks. The decline in FVC was significantly greater in the placebo group than in the group treated with nintedanib (−187.8 mL/year versus −80.8 mL/year; 95%CI 65.4–148.5, *p* < 0.001). The adverse event profile associated with nintedanib was acceptable and similar to the one observed in IPF. According to a subgroup analysis of the INBUILD trial by ILD diagnoses, nintedanib reduced the rate of disease progression equally across the five ILD subgroups, regardless of the underlying diagnosis [52]. Safety and tolerability were also similar across diagnostic subgroups.

Pirfenidone has so far been investigated for the treatment of progressive fibrosing unclassifiable ILD in a phase 2 trial involving 253 patients [15]. The primary endpoint was the mean change in FVC measured over 24 weeks with home spirometry. However, the primary endpoint could not be properly evaluated due to the variability and unreliability of the values obtained from home spirometry. Although the trial suggested a potential benefit for pirfenidone, conclusions were limited by the study design. The FVC changes over 24 weeks were −87.7 mL and −157.1 mL with pirfenidone and placebo, respectively. The most common treatment-related adverse events were gastrointestinal disorders, fatigue and rash.

Our understanding of the potential of antifibrotics for the treatment of progressive fibrosing ILDs is likely to improve soon, as a number of clinical trials with nintedanib and pirfenidone are ongoing [53]. Other agents are also under investigation. Pamrevlumab, a monoclonal antibody against connective tissue growth factor, which plays an important role in fibrosis, has been shown in the phase 2 trial PRAISE to significantly reduce the progression of IPF compared with placebo, resulting in a lower proportion of patients with disease progression compared with placebo at 48 weeks of treatment (10.0% versus 31.4%, respectively; *p* = 0.013) [54]. Pamrevlumab was well tolerated with no significant differences from placebo in the adverse event profile. Galectin-3 is another potential target under investigation as antifibrotic therapy in IPF. Galectin-3 is a pro-fibrotic ß-galactoside-binding lectin implicated in the pathogenesis of IPF and IPF exacerbations [55,56]. A phase 1/2a study with Td139, an inhaled inhibitor of galectin-3, in healthy subjects and patients with IPF has provided promising results [56]. Td139 was well tolerated and shown to significantly inhibit galectin-3 expression in alveolar macrophages compared with placebo and to reduce serum levels of other potential biomarkers of IPF progression.

## 5. Adoption of New Drugs in Clinical Practice and Treatment Optimization

Key issues for improving the treatment of IPF and other progressive fibrosing ILDs include multidisciplinary management of patients from diagnosis to treatment and follow up, early referral to specialty centers, close monitoring for disease progression and the continuous update of information and management strategies as the field is rapidly evolving. Evidence from a prospective cohort study in IPF patients suggests that delayed referral to tertiary care centers is associated with increased mortality regardless of IPF severity [57]. According to the results of this study, the median delay (i.e., time from the onset of dyspnea to first evaluation at a tertiary center) was 2.2 years, and the median follow-up time was 1.1 years [57].

There is an urgent need for guidelines helping clinicians in the management of ILD patients with the new treatment options. As pointed out in a series of suggestions on how to improve future guidelines for the diagnosis and management of IPF, recommendations for progressive fibrosing ILD should not be added to IPF guidelines as this may create additional confusion [23]. Thus, guidelines for the treatment of progressive fibrosing ILD should be addressed in a separate document. Moreover, formulations such as the “conditional recommendation” used in the 2015 ATS/ERS/JRS/ALAT guideline for IPF treatment should be avoided and replaced by the concept of “case-by-case evaluation” [23].

The INBUILD trial has shown that when the progressive fibrosing phenotype develops, a definite diagnosis of the underlying ILD is not required to initiate antifibrotic therapy with nintedanib and that a working diagnosis is sufficient [52]. This observation allows us to confirm that the attitude of splitters for different diagnostic subgroups does not seem to be key for optimally managing progressive fibrosing ILD; therefore, it is perhaps better to be lumpers. Hence, it seems to be an innovative aspect to underline that, more than splitters, pulmonologists who deal with ILD must learn to become smart lumpers once IPF is excluded, in which case being an analytical splitter is still fundamental. Data further supporting this therapeutic strategy comes from studies showing either previous or simultaneous use of anti-inflammatory and/or immunosuppressant drugs not only appears not contraindicated but also appears useful when used in combination with antifibrotics [58,59]. Therefore, on the basis of published data concerning all progressive ILD pathologies, it is not necessary to classify and differentiate the individual pathologies, however, a method to define the progressive behavior of the ILD as early as possible should be sought. Making therapeutic decisions based on a working diagnosis will increase the access of ILD patients to effective therapies. At the same time, however, many clinicians may be reluctant to use this approach [37,52]. 

The use of combination therapy that combines, for example, antifibrotic and immunomodulatory agents may further improve the treatment of progressive fibrosing ILDs. Subgroup-analyses of the INBUILD and SENSCIS trials by immunomodulator use at baseline have suggested that nintedanib can be used in combination with glucocorticoid [58] and mycophenolate [59] without affecting the efficacy of nintedanib on disease progression. The potential of the combination of nintedanib with the novel antifibrotic Td139 (galectin-3 inhibitor) has also been suggested recently [60].

In the justification of a progressive fibrotic phenotype, the clinical and biological rationale must complement therapeutic decisions, with improved understanding on how to best define disease progression and, ideally, how to predict its occurrence prior to actual progression of the utmost importance [61]. However, there remain unmet clinical needs regarding the evaluation and management of the progressive fibrotic phenotype [62]. As our understanding of the pathogenesis of progressive fibrosing ILDs increases and biomarkers of disease progression become available, it will be possible to further improve treatment towards precision medicine and the targeted treatment of individual members of the ILD group [8].

## 6. Conclusions

Our current understanding of the pathogenesis of progressive fibrosing ILDs is limited, and treatment guidelines, other than for IPF, are lacking. However, the results of trials investigating antifibrotic drugs in patients with fibrosing ILDs other than IPF, which have shown a reduction in the rate of disease progression, are considered a breakthrough in the field of ILD. Importantly, the INBUILD trial demonstrated that a definite diagnosis of the underlying ILD was not required to initiate antifibrotic therapy when the progressive fibrosing phenotype develops and that a working diagnosis was sufficient. Therapeutic decisions based on a working diagnosis are likely to increase the access of ILD patients to highly effective therapies and ultimately improve long-term outcomes. Ideally, an increasing understanding of the pathogenesis of progressive fibrosing ILDs, a clear definition of a progressive fibrosing phenotype and the identification of validated biomarkers to predict disease progression may direct treatment towards more precise and targeted options. Indeed, ongoing clinical trials with nintedanib and pirfenidone, as well as other novel antifibrotic agents under investigation, are expected to advance our understanding of the therapeutic potential of antifibrotics for the treatment of progressive fibrosing ILDs. Ideally, it is expected that the adoption of new drugs in clinical practice alongside treatment optimization will improve the management of patients with these rare conditions.

## Figures and Tables

**Table 1:** Suggested definitions of the progressive fibrosing phenotype (reproduced with permission from George et al. 2020 [40]).

Definitions
1. Relative decline of ≥10% in FVC over 24 months despite treatment.
2. Relative decline of ≥5% in FVC with decline in diffusing capacity of the lung for carbon monoxide of ≥15% over 24 months despite treatment.
3. Relative decline of ≥5% in FVC with increased fibrosis on HRCT ^1^ over 24 months despite treatment.
4. Relative decline of ≥5% in FVC with progressive symptoms over 24 months despite treatment.
5. Progressive symptoms with increased fibrosis on HRCT ^1^ over 24 months despite treatment.

Abbreviations: FVC, forced vital capacity; HRCT, high resolution computed tomography. ^1^ As assessed by an expert thoracic radiologist.
